# Navigating uncertainty: pre-diagnostic tuberculosis experiences and care-seeking in Blantyre, Malawi

**DOI:** 10.3389/fpubh.2026.1913596

**Published:** 2026-10-27

**Authors:** Robina Semphere, Madalo Mukoka, Takondwa C. Msosa, Hussein H. Twabi, Peter MacPherson, Rufaro Mushonga, Marriott Nliwasa, Moses K. Kumwenda

**Affiliations:** 1 Chichiri Centre for Health Research and Training, Kamuzu University of Health Sciences, Blantyre, Malawi; 2 School of Health and Wellbeing, University of Glasgow, Glasgow, United Kingdom; 3 Department of Population Health, London School of Hygiene and Tropical Medicine, London, United Kingdom; 4 Department of Epidemiology and Biostatistics, School of Global and Public Health, Kamuzu University of Health Sciences, Blantyre, Malawi; 5 Department of Global Health, Amsterdam University Medical Centres, Amsterdam, Netherlands; 6 Institute of Life Course and Medical Sciences, University of Liverpool, Liverpool, United Kingdom; 7 Department of Mental Health, Faculty of Medicine and Health Sciences, University of Zimbabwe, Harare, Zimbabwe; 8 School of Public Health, University of the Witwatersrand, Johannesburg, South Africa; 9 Institute of Resilient Health Systems, Department of Clinical Sciences and Department of International Public Health, Liverpool School of Tropical Medicine, Liverpool, United Kingdom

**Keywords:** care-seeking, diagnostic delay, Malawi, patient-centered care, tuberculosis

## Abstract

**Introduction:**

Tuberculosis (TB) remains a major public health challenge in Malawi. While diagnostic pathways are well established, less is known about how people experience repeated care-seeking and diagnostic uncertainty before receiving a TB diagnosis.

**Objective:**

To explore pre-diagnostic TB experiences and care-seeking among adults with TB and caregivers of children with TB in urban Blantyre, Malawi.

**Methods:**

We conducted in-depth interviews with 14 participants in urban Blantyre, Malawi (five male patients, five female patients, and four female caregivers acting as proxies for pediatric TB patients under 11 years). Data were coded deductively and inductively, then analyzed using a framework approach. Social Cognitive Theory informed the interview guide, and Candidacy Theory informed interpretation of access to TB investigation across multiple healthcare encounters.

**Results:**

Participants described prolonged, distressing pre-diagnostic journeys, with early symptoms of TB frequently misattributed to malaria or minor respiratory conditions. As symptoms persisted, participants reported repeatedly seeking care, often attending health facilities multiple times before TB testing was initiated. These encounters did not provide immediate diagnostic resolution and represented participant-reported opportunities for earlier TB investigation. Participants’ social networks shaped decision-making along the pathway, occasionally reinforcing misattribution but often prompting escalation to formal care. Repeated unresolved encounters imposed substantial economic burden on participants and their families, including loss of income, business disruption, and increased reliance on family support.

**Conclusion:**

In urban Malawi, participants described TB diagnostic pathways shaped by symptom uncertainty, social and material circumstances, and repeated healthcare encounters without timely diagnostic resolution. The findings highlight the potential value of reassessing persistent or worsening symptoms during repeat encounters. Strengthening such reassessment may support integrated, patient-centered TB care and reduce avoidable socioeconomic burden.

## Introduction

Tuberculosis (TB) remains a major public health challenge globally, and remains the leading cause of death globally from a single infectious agent (1, 2). According to the Global Tuberculosis Report 2025, an estimated 10.7 million TB cases and 1.23 million deaths were reported in 2024, yielding a case fatality rate of 11.5% (3). Low- and middle- income countries account for over 80% of cases and deaths (4). The Global Plan to END TB, 2023–2030, is a comprehensive strategy to end TB by 2030. The report suggests that if the current public health efforts to combat TB remain unchanged, an additional 43 million people could develop TB, resulting in an additional 6.6 million deaths by 2030 (5). Achieving TB elimination will not only require improving diagnosis and reduced treatment regimen duration, but critical examination of how care is delivered and experienced.

Between 2014 and 2015, WHO and United Nations Member States united to end the TB epidemic by adopting the World Health Organization’s (WHO) End TB Strategy and the United Nations Sustainable Development Goals (6). One of the suggested priority actions of the WHO End TB Strategy is the implementation of Pillar 1, which mandates integrated, patient-centered care and prevention (7). Implementing a patient-centered approach requires more than health system strengthening, it demands a clearer understanding of how TB is experienced, particularly from the perspective of those navigating through the care-cascade and how diverse communities and contexts they come from shape their health seeking behavior. Without a deep understanding of patients lived experiences through their care-seeking pathways, commitments to patient-centered care risk remaining aspirational rather than transformative.

A substantial body of literature has examined “diagnostic delay,” in TB, often defining it as a measurable time interval between symptom onset and treatment initiation. While some have provided a general description of diagnostic delay in patients (8–10), others have distinguished between the interval from symptom onset to first healthcare contact (patient delay), and the interval from first contact and treatment initiation (health system delay) (11–17). This is not merely a technical distinction; it is analytically and programmatically crucial. Patient delay reflects processes occurring at individual, household and community levels. In contrast, health system delays reflect processes within facilities and health systems. Failing to distinguish these intervals risks masking where responsibility lies and misdirecting interventions.

To date, much of the evidence on diagnostic delay has been quantitative, identifying determinants such as sociodemographic characteristics, knowledge, stigma, education, and geographical access (11, 17–19). Some studies have attributed the delay to late presentation (7, 17, 18, 20) while other studies attributed this to health system factors (21–25). Although these studies help to estimate the magnitude of delayed TB diagnosis and identify high-risk groups, they tend to conceptualize delay as an outcome rather than as a dynamic process (8, 18, 19, 21–23, 25, 26). Qualitative inquiry allows us to examine how these delays are produced, negotiated, and sustained within everyday life and sheds light on how structural constraints intersect with individual agency, and how health system encounters may reinforce or alleviate delay. Without a deeper understanding of how patients interpret and respond to illness, how their community and prior experiences with care influence subsequent decisions, interventions risk being generic and insufficiently responsive to the lived realities of patients.

Emerging qualitative and mixed-method studies have begun to explore patient perspectives on tuberculosis diagnostic delay across diverse settings. A qualitative study in Portugal highlighted how limited TB literacy, socioeconomic vulnerability, stigma, and prior healthcare experiences shaped patients’ interpretations of symptoms and their timing of care-seeking (27). Similarly, a rapid review of evidence from low-burden, high-income countries identified both patient-level and health service determinants of delay, but noted that qualitative evidence remained limited and often restricted to specific subgroups (28). In Bangladesh, a large mixed-method study estimated substantial patient delay and identified predictors such as self-treatment and diabetes, with qualitative findings underscoring symptom normalization, financial hardship, and initial reliance on informal providers (29). Collectively, these studies advance understanding of factors associated with delay, yet they largely frame delay as a measurable outcome or a set of determinants, rather than examining how delay unfolds as a dynamic, socially embedded process co-produced through interactions between patients, informal care networks, and health systems, particularly within high-burden urban African contexts.

Despite the growing body of evidence on diagnostic delay, there remains limited qualitative research examining how patients experience and navigate the TB diagnostic pathway within urban primary care settings in high-burden contexts. In settings such as urban Blantyre, Malawi, where patterns of health facility bypassing, self-referral, and community-based screening intersect with routine primary care delivery, understanding how patients interpret symptoms, choose care pathways, and encounter health services is essential for designing responsive interventions. This study therefore sought to explore the pre-diagnostic experiences of patients and caregivers to patients with TB.

## Methods

### Study design and setting

This cross-sectional qualitative design explored participants’ experiences from initial symptom recognition through the period immediately preceding TB diagnosis across five primary healthcare facilities in urban Blantyre, Malawi. Study sites included Bangwe Health Centre, Gateway Clinic, Limbe Health Centre, Makhetha Health Centre and Chilomoni Health Centre. The study focused on how participants interpreted symptoms, sought advice and care, navigated multiple healthcare encounters, and experienced the consequences of diagnostic uncertainty.

### Research team and reflexivity

The research team consisted of clinicians and public health researchers working in TB-related settings in Malawi. A total of three interviewers were involved during the recruitment and data collection process (RS- a clinical epidemiologist and PhD candidate with experience conducting qualitative interviews, CC- a research assistant with qualitative training and experience, OM - a research assistant with qualitative training and experience). RS was responsible for the development of concepts, protocol and data collection tools, ethics submission, data collection, coding, analysis and manuscript development. CC and OM were responsible for data collection and coding. Two of the interviewers were male (CC and OM) and one was female (RS), and the female interviewer was present during all interviews. Reflexive discussions were held to consider how clinical training and prior engagement with TB programs could influence interpretation. At least one observer was present during each interview to take notes and provide additional perspective when discussing preliminary reflections from interviews. There was no relationship established between researchers and participants prior to recruitment. Participants were informed about the aims of the study and were told that their participation could potentially contribute to improving TB care and services. Researchers also disclosed their roles at the beginning of the interview and clarified that they were not employed by the health facilities, to reduce the potential influence of participants’ responses based on perceived associations with facility staff.

### Participant selection

Participants were purposively selected from five primary healthcare facilities to capture variation in TB diagnostic and care-seeking trajectories. Makhetha Health Centre, Gateway clinic, Limbe Health Centre, Chilomoni Health Centre, and Bangwe Health Centre were selected based on routine program data indicating differing patterns of health-facility bypassing. Specifically, the facilities varied in the proportion of patients residing within each facility’s catchment area who bypassed that facility to seek care elsewhere. Including facilities with differing bypassing patterns enabled exploration of how participants’ experiences of symptom interpretation, care-seeking, facility choice, and movement between services shaped pathways to TB diagnosis. Gateway clinic was additionally selected because it frequently receives patients who bypass a primary facility and self-refer to the public tertiary health facility, Queen Elizabeth Central Hospital, and are subsequently referred back to Gateway clinic. The clinic also serves as a base for a community tuberculosis mobile van, primarily used for community-based TB screening and diagnosis. This enabled the identification of patients diagnosed through mobile vans, with support from the TB officers operating both at Gateway clinic and the van.

Participant recruitment was conducted with the assistance of TB officers at the selected facilities. As part of the routine TB program responsibilities, TB officers maintain records of patients receiving TB care and have ongoing contact with patients attending follow-up and medication refill visits. They therefore provided the most appropriate point of contact for identifying participants who met the eligibility criteria. TB officers approached potentially eligible patients attending routine follow-up visits and contacted other potentially eligible patients by telephone.

We sought variation across participant role (adult patient or caregiver of a child with TB), treatment status at interview, mode of diagnosis and TB type. Eligible participants therefore included adults receiving TB treatment, individuals who had completed treatment, caregivers of pediatric TB patients younger than 11 years, individuals diagnosed through community-based screening using the TB van, and patients with pulmonary TB or extrapulmonary TB. This approach to sampling intended to maximize experiential variation relevant to the research question rather than statistical representativeness.

### Data collection

Data were collected from 3rd -18th June 2025. All interviews were conducted in Chichewa, the predominant language spoken in the study area. The in-depth interviews each lasted 40 to 90 min. No repeat interviews were conducted. In-depth interviews explored participants’ experiences from initial symptom recognition through to TB diagnosis. The RS developed semi-structured interview guides that included probes on perceived causes of symptoms, advice from family and friends, care-seeking pathways (including use of other facilities, medications, or traditional remedies), beliefs and knowledge about TB, and the social, economic, and psychological burden of illness. Interview locations were selected according to participants’ preferences and included participants’ homes, TB health facilities, and the Kamuzu University of Health Sciences campus. No one other than the participant and members of the research team was present during the interviews, thereby protecting privacy. Open-ended questions were used and adapted subsequent questions and probes based on the participants’ answers and emerging issues. All participants provided written consent for audio-recording, and digital audio recorders were used to ensure accurate capture of interview data. Recruitment continued until the research team agreed during ongoing debriefing that subsequent interviews were no longer generating substantive new insights relevant to the study objectives. Transcripts were not returned to participants for comments or corrections. In accordance with qualitative approaches that prioritize an in-depth capture of narratives, the study emphasized iterative team-based coding, reflexive discussions, and comparison across participants to ensure credibility. Multiple researchers were involved in coding, and findings were grounded in verbatim quotations to maintain close alignment with participants’ accounts.

### Data analysis

Data analysis followed an iterative, theoretically informed framework analysis combining deductive and inductive coding (Table 1). Social cognitive theory informed the development of the interview guide and provided an initial orientation to individual and social processes influencing symptom interpretation and care-seeking (30). In particular, the interview guide explored how participants prior experiences, knowledge, beliefs and social influences shaped individual interpretation of symptoms and decisions about when and where to seek care. The theory highlights key constructs such as observational learning, self-efficacy, and outcome expectations, and also recognizes that behavior is influenced by reciprocal interactions between personal factors, social context, and environmental constraints.

**Table 1:** Relationship between theoretical perspectives, coding processes, framework analysis, and final interpretive synthesis.

Analytic component	Function in the study	Examples of concepts
Social cognitive theory	Informed interview guide and initial analytic orientation	symptom interpretation, prior experience, social influence, outcome expectations
Deductive coding	Captured study objectives and domains explored in the interview guide	Symptoms and perceived causes, care-seeking, TB knowledge and beliefs, social support, economic burden
Inductive coding	Captured concepts emerging from Captured concepts emerging from participant and caregiver narratives	Repeated attendance, diagnostic uncertainty, escalating concern, recurrence of symptoms
Framework analysis	Supported comparison of pre-diagnostic trajectories within and across participants	Repeated encounters, referral pathways, evolving symptom interpretations, social and economic consequences
Candidacy theory	Applied at the interpretive stage to examine access to TB investigation across healthcare encounters	Recognition of need, navigation of services, provider adjudication, negotiated access
Final synthesis	Integrated participant, social, and healthcare-access processes	Diagnostic loop, participant-reported missed opportunities for earlier TB investigation, negotiated candidacy

During analysis, repeated accounts of healthcare interactions without diagnostic resolution indicated that individual and social explanations were insufficient to account for participants’ experiences. Candidacy Theory was therefore introduced as an additional interpretive framework to examine how recognition and access to TB investigation were negotiated across successive healthcare encounters (31, 32). The theory outlines stages through which individuals’ eligibility for care is constructed, including identification of need, navigation of services, and adjudication by healthcare providers. It also emphasizes how structural factors and provider–patient interactions can facilitate or constrain recognition of patients as legitimate candidates for care. This theory conceptualizes access to healthcare as a negotiated process between patients and health services, to fully examine how access to diagnosis is constructed across multiple healthcare encounters. This iterative integration of theoretical perspectives aligns with qualitative approaches that allow analytical frameworks to evolve as findings emerge.

A hybrid deductive-inductive coding approach was used. Initial codes were formulated by one researcher (RS) prior to data collection based on study objectives, relevant literature and the domains explored in the interview guide. Following data collection, three researchers (RS, CC, OM) independently reviewed transcripts after data collection to refine the coding framework based on the emerging concepts (Table 2). Themes were derived from the findings that emerged from the data. All recordings were transcribed and translated into English using Express Scribe transcription software. Data was systematically coded and managed using NVivo 14 (QSR International) to support thematic analysis.

**Table 2:** Analytic trail from initial NVivo codes to results subsections, overarching themes, and interpretive constructs.

**Overarching theme**	**Results subsection**	**Principal NVivo codes**	**Analytic pattern evident in the results**	**Interpretive construct/framework**
Symptom recognition and interpretation	Knowledge, beliefs, and perceptions of TB	Knowledge about TB; beliefs and perceptions about TB; TB contacts	Knowledge ranged from accurate understanding to misconceptions about TB transmission, risk, severity, and extrapulmonary disease. TB knowledge was often socially mediated or developed during the illness journey.	Social Cognitive Theory: knowledge, social learning, and expectations shaped early appraisal of illness.
Symptom recognition and interpretation	Symptom recognition and misattribution: Interpreting illness through familiarity and fear	Symptoms and perceived causes; comorbidities; coping with disease; motivation	Participants initially attributed symptoms to familiar illnesses, including malaria, asthma, stomach problems, worms, abscesses, or a non-specific cough. Reappraisal followed persistence, recurrence, functional decline, or worsening symptoms.	Early uncertainty and evolving recognition of the need for care.
Care-seeking patterns	Social advice and lay consultation: Navigating illness through social networks	Family and social support; advice and guidance; beliefs and perceptions about TB; coping with disease	Social networks influenced how symptoms were interpreted and how care was sought. Advice could promote household remedies or traditional healing, but also formal care, referral, transport, finances, and accompaniment.	Social Cognitive Theory: social influence; Candidacy Theory: family and social resources enabled or constrained navigation.
Influences on access to diagnosis	Repeated care-seeking without diagnostic resolution: Cycles of hope and frustration	Healthcare access before TB diagnosis; quality of initial care; symptoms and perceived causes; comorbidities; motivation	Participants described multiple healthcare encounters, alternative tests, empirical treatment, temporary relief or continued illness, symptom recurrence, and re-attendance at the same or another facility.	**Diagnostic loop**; participant-reported missed opportunities for earlier TB investigation.
Influences on access to diagnosis	Repeated care-seeking without diagnostic resolution: Cycles of hope and frustration	Healthcare access before TB diagnosis; quality of initial care; accessibility/convenience; transportation; motivation	Despite repeated presentation and active navigation of services, TB investigation was commonly delayed until treatment failed, symptoms worsened, referral occurred, or another diagnostic service was reached.	Candidacy Theory: repeated navigation did not consistently result in recognition and adjudication as a candidate for TB investigation.
Influences on access to diagnosis	Economic, psychological, and emotional burden	Burden/impact of disease; cost; transportation; coping with disease; family and social support	Repeat attendance and unresolved illness caused transport expenditure, income and business losses, school interruption, reliance on others, fear, frustration, and exhaustion.	Cumulative household consequences of unresolved care-seeking and delayed diagnostic resolution.

Coded data were subsequently charted in a framework matrix organized around participants’ pre-diagnostic trajectories. Regular debriefing meetings were conducted to resolve coding discrepancies, refine codes and ensure consistency. Codes were grouped to identify themes and subthemes, and illustrative quotations were selected to preserve patients’ voices and support our interpretation of the findings. Candidacy Theory was used primarily at this interpretive stage to examine how repeated healthcare encounters shaped or constrained recognition of participants as candidates for TB investigation. Reflexivity was maintained throughout the analysis to account for the influence of researchers’ perspectives on interpretation.

The study focused specifically on the early/pre-diagnostic segment of the TB care cascade, from symptom recognition and care-seeking through to diagnostic resolution. Participant characteristics were therefore considered as sources of variation in these pre-diagnostic experiences rather than as predefined analytical subgroups. Given the small sample and the study’s focus on processes across care-seeking trajectories, formal subgroup comparisons by treatment status, TB type, diagnostic route, or participant role were not undertaken. These characteristics were retained in the framework matrix and considered during cross-case interpretation. Experiences relating primarily to subsequent stages of the TB care cascade, including treatment-related experiences, and side effects, were outside the scope of the present analysis.

An analytic trail was maintained through successive versions of the coding framework, framework matrices, team discussions, and documentation of coding decisions and theme development (Table 2). The analytical process moved from initial descriptive codes to grouped categories, subthemes, and overarching themes.

### Ethical considerations

The studies involving human participants were reviewed and approved by the College of Medicine Research and Ethics Committee (COMREC; approval number P.01/25–1,383). Additional approval was obtained from the Blantyre District Health Office. Written informed consent was obtained from all adult participants before enrolment. Caregivers provided written informed consent for their own participation in interviews concerning the pre-diagnostic TB experiences of their children; no children were interviewed. Participants provided written consent for audio-recording and were informed that de-identified interview data and anonymized quotations could be used in publications.

## Results

A total of 18 participants were approached by the study team, and four individuals did not participate. The four individuals did not participate because they could not be reached before their scheduled interview dates. Participants included five male patients, five female patients and four female caregivers of patients who started TB treatment between November 2024 to June 2025 (Table 3). The age of the patients ranged from 21 to 53 years. Most of the respondents were married (9/14, 64.3%). Regarding educational attainment, 4/14 (28.6%) attended part of primary school education, 9/14 (64.3%) attended secondary school education, and 1/14 (7.1%) completed tertiary level education. In terms of occupation, 11/14 (78.6%) owned small scale businesses, one was employed (7.1%), one performed piece work for a living (7.1%) and the remaining was a student (7.1%). Regarding mode of diagnosis, 9 of 14 participants (64.3%) were diagnosed at primary healthcare facilities, 3 (21.4%) through community mobile TB vans, and 2 (14.3%) at a tertiary-level health facility before being referred back to primary facilities for continuation of care. During the interview, 10/14 (71.4%) were on TB treatment, and 4/14 (28.6%) had completed their treatment regimen. The dominant religion was Christianity (100%).

**Table 3:** Demographic characteristics of study participants (*n* = 14).

Characteristic	*N* (% or range) Total = 14
Sex	
Female	9 (64.3)
Male	5 (35.7)
Age (Years)	
Median (Range)	35 (21–53)
Participant type	
Patient	10 (71.4)
Caregiver	4 (28.6)
Marital status	
Married	9 (64.3)
Single	5 (35.7)
Education level	
Primary education	4 (28.6)
Secondary education	9 (64.3)
Tertiary education	1 (7.1)
Occupation	
Small-scale business owners	11 (78.6)
Formally employed	1 (7.1)
Piece work	1 (7.1)
Student	1 (7.1)
Mode of diagnosis / Referral	
Primary health facility-based diagnosis	9 (64.3)
Community mobile van diagnosis	3 (21.4)
Referral from tertiary facility	2 (14.3)
TB treatment status at interview	
Currently on treatment	10 (71.4)
Completed treatment	4 (28.6)
Type Of TB experience	
Pulmonary Tb	12 (85.7)
Extrapulmonary TB	1 (7.1)
Relapse TB	1 (7.1)
Distance To TB facility	
≤ 30 min Walk	6 (42.9)
>30 min walk	6 (42.9)
Distance reported in kilometers	2 (14.3)

### Overview of themes

Patients’ pre-diagnostic TB experiences yielded three overarching themes: symptom recognition and interpretation, care-seeking patterns, and influences on access to diagnosis. These themes capture how participants and caregivers understood early TB symptoms, the factors influencing when, where, and from whom they sought care; and the social, economic and health-system conditions that shaped progression towards diagnosis. The sections below present the subthemes contributing to each overarching theme. Across these findings, recurrent accounts of persistent symptoms, repeated healthcare attendance, alternative treatment or diagnostic pathways, and delayed TB investigation informed the interpretation of a diagnostic loop and participant-reported missed opportunities for earlier TB investigation. The analytic relationship between initial codes, subthemes, overarching themes, and theoretical interpretation is shown in Table 2.

### Symptom recognition and interpretation

#### Knowledge, beliefs, and perceptions of TB

Participants’ knowledge and perceptions of tuberculosis varied considerably, with a mixture of accurate understanding, misconceptions, and evolving beliefs resulting from lived experience and interactions with the health system. Some understood TB as a contagious but treatable condition, often describing as transmissible through “wind, air or breath.” For others, TB was associated with age, obesity, death, severe weakness, social behavior, or HIV, intensifying fear and emotional distress. Notably, most participants (apart from a caregiver on TB treatment, whose infant was also diagnosed) did not know how TB was acquired, although one previously had TB and was able to recognize the symptoms. Knowledge of TB was often acquired progressively through repeated interactions with health services, rather than existing prior to illness. Some participants’ knowledge was socially mediated through family and friends, shaping both expectations and care-seeking decisions. Notably, all participants who had extrapulmonary TB were unaware that the disease can affect organs beyond the lungs;

*“We were all surprised because there was no one at home who ever had TB. TB is spread through the air when you are close to a TB patient.”* (IDI_A7R6, female, 28, caregiver, child 1 year 9 months, on treatment).

*“People describe the disease as deadly… So, I was scared as I had little to no knowledge regarding it… Some people told me that TB is a disease for old people which was scary because I was wondering how I got sick if that is the case…”* (IDI_X4N2, female, 21, patient on treatment).

*“We learn about TB from our communities during funerals. most people think that TB is transmitted through “naughty” behavior*” (IDI-N0C3, female, 53, completed treatment).

*“I knew only about pulmonary TB… I wish I had known about the other types of TB… I knew about pulmonary TB only and this was my first time to know that spine TB exists, and I do not know what really causes that kind of TB.”* (IDI_M3P9, female, 25 years, caregiver, child 9 years, completed treatment).

#### Symptom recognition and misattribution: interpreting illness through familiarity and fear

Participants’ illness journeys usually began one or more of gradually worsening, persistent and/or ambiguous symptoms. They described prolonged cough, breathlessness, chest pains, dizziness, confusion, chills, fever, vomiting, neck swelling, and a progressive loss of strength that eventually interfered with walking or working. Their first impression of their illness was familiar and socially recognizable illness categories, including malaria, asthma, or “just a cough.” One participant recalled, “*I was feeling like I had asthma… I was having difficulties in breathing… I did not think it was TB*,” (IDI_H9L8, female, 44, patient on treatment, mobile van) while another explained, “*I used to vomit when I ate anything… I thought it was just a stomach problem*.” (IDI_C2V7, male, 45, patient completed treatment).

At the early stages of disease, symptoms were often normalized or minimized by patients, especially when they temporarily improved using symptom-related medication. Several patients experienced moments of partial relief, followed by symptom recurrence, reinforcing uncertainty and delaying suspicion of a more serious diagnosis. Participants described bodily functional decline, such as increased breathlessness when walking and inability to work. At this point, the symptoms were re-evaluated as abnormal. One participant explained, “*When I compared how I was to how I used to be; I saw that my body wasn’t the way it was before*.” (IDI_PLT_X9A3, male, 28, patient on treatment). Most patients recognized that something was wrong when additional symptoms emerged, or previous symptoms remained persistent, progressively worsened, or impeded their regular activities.

For caregivers, early symptoms in their children were often subtle and unspecific, and were attributed to asthma, worms, abscesses or a general cough. They often noted bodily changes, such as persistent coughing with thick sputum, vomiting after feeding, swelling of limbs, weight loss, weakness, and reduced appetite, while struggling to understand their cause. A caregiver explained, “*We were saying that it was asthma. I told my grandmother who told my Auntie that it was asthma because the child was having breathlessness intermittently.”* (IDI_A7R6, female, 28, caregiver, child 1 year 9 months, on treatment) Another mother recalled, *“My child had started with a lump on the cheek… we did not understand what it was at first.”* (IDI_J1F5, female, 43, caregiver, child 10 years, on treatment) She described attempting to drain what was thought to be an abscess and repeated hospital visits with minimal resolve. As illness persisted, the children’s functional decline became increasingly concerning, with caregivers describing episodes of collapse, inability to walk, and involuntary urination and defecation. This decline prompted fear and urgency, even if TB was not yet suspected. Caregivers also reported repeated episodes of seeking care and symptom relief, only for the symptoms to return or worsen, further delaying the recognition of TB. For many, this cycle would last from weeks to months, with caregivers of young children taking initiatives to make sense of the deterioration and making effort to navigate diverse care pathways until escalation or acute events resulted in referral to higher-level facilities where TB was diagnosed.

### Care-seeking patterns

#### Social advice and lay consultation: navigating illness through social networks

Decisions about care-seeking were rarely made in isolation as social networks were frequently involved in discussions and interpretations of the patients’ illnesses. This social network community included family members, partners, neighbors, teachers, employers, and friends. These networks functioned as sources of sense-making, naming symptoms, comparing past experiences and advice about what actions were appropriate;

*“From Machinga, they (family) said I should go to Traditional healers for them to cut me (for medicinal purposes “Kundichekera”).* (IDI_PLT_T8F1, female, 40, patient on treatment).

*“My brother’s wife heard that I was sick… she asked her friend to give me a Ceftriaxone injection at home…Even after 13 days, there was no improvement.”* (IDI-N0C3, female, 53, completed treatment).

Lay consultation often drew experiences from previous familiar symptoms and locally available remedies. While such advice demonstrated concern, it sometimes reinforced misattribution and prolonged experimentation of alternative interventions outside formal healthcare because of the perceived distrust in the ability of the health system to manage the condition. Recommendation of these interventions positioned illness as something that could first be managed within the household or community.

*“I told my grandmother who told my Auntie that it was asthma because the child was having breathlessness intermittently.”* (IDI_A7R6, female, 28, caregiver, child 1 year 9 months, on treatment).

*“They told me to use guava leaves as a remedy and I will be well but that I wasn’t. Some relatives told me to drink a mixture of flour and water …. I tried all that, but it did not stop… others would tell me to drink a mixture of ground charcoal and water.”* (IDI_R8W4, male, 31, completed treatment).

At the same time social networks acted as powerful catalysts for positive health-seeking behavior, especially when symptoms worsened, disrupted normal functioning, or if the patient was in contact with someone who could enforce positively enabling behavior. Family members intervened when illness became visibly severe. Beyond providing understanding and suggestions, patients’ social networks provided practical support that made care-seeking possible. Relatives and neighbors provided transport, accommodation, finances and accompaniment to the hospital. Others helped in navigating the health system, advising against lower-level facilities or private providers in favor of public hospitals perceived to offer more effective treatment;

*“People at home suggested that we could have been doing a wrong thing giving the child traditional medicine which could end up into something bad.”* (IDI_A7R6, female, 28, caregiver, child 1 year 9 months, on treatment).

*“My brother told me to go to Limbe health centre…one of my neighbors has a motorcycle and I booked the motorcycle to take me to the hospital in the morning…he charged me fairly since we are neighbors”* (IDI-N0C3, female, 53, completed treatment).

*“It reached a point that my family told me to request for a referral letter to Queen Elizabeth Central Hospital (Queen’s)…I felt that I could not decide on behalf of healthcare workers by requesting a referral letter to Queen’s. But one day the child woke up unwell and very weak, and we then decided to go straight to Queen’s.”* (IDI_A7R6, female, 28, caregiver, child 1 year 9 months, on treatment).

### Influences on access to diagnosis

#### Repeated care-seeking without diagnostic resolution: cycles of hope and frustration

Most participants described multiple encounters with the health system before receiving a tuberculosis diagnosis, none of which resolved their illness. These visits involved either testing for other illnesses, reassurance or symptomatic treatment. Participants described alternating periods of optimism and disappointment: hope that treatment would resolve symptoms, followed by frustration when symptoms persisted or worsened. These hospital visits often resulted in temporary relief, followed by recurrence. Each return to care represented both persistence and renewed uncertainty. For some participants, referrals between facilities further extended the diagnostic journey, while for others the absence of timely diagnostic testing intensified physical deterioration. Importantly, most participants did not disengage from care; instead, they repeatedly re-entered the health system, often at significant personal cost. These repeated encounters accumulated emotional fatigue, financial strain, and physical exhaustion, reinforcing a sense of being caught in an unresolved diagnostic loop rather than progressing along a clear pathway to care.

Across accounts, this pattern formed the empirical basis for the interpretive construct of a diagnostic loop. Participants described a recurring sequence in which persistent or worsening symptoms prompted an initial or repeat healthcare visit; the encounter resulted in alternative testing, an alternative explanation, reassurance, or symptomatic treatment; symptoms then persisted, worsened, or returned; and participants subsequently sought care again, either at the same facility or elsewhere. The loop therefore reflected repeated attempts by participants and caregivers to obtain relief and diagnosis, rather than passivity or disengagement from care.

“*I was going to different hospitals, they would say, ‘Let us test for Malaria…can I test you for HIV?’…They would give me anti-biotics… and when I would finish the dose, it all would come back. I was desperate to breathe normally so I was fine with them testing me for any sickness”* (IDI_H9L8, female, 44, patient on treatment, mobile van).

*“They just kept telling me to go get him tested for Malaria. Whenever he was Malaria negative, he was given Amoxicillin or Bactrim and we were sent home…for 5 visits”* (IDI_A7R6, female, 28, caregiver, child 1 year 9 months, on treatment).

*“We did not receive any treatment initially because they were still trying to figure out what was wrong with her… We spent a month and some weeks in the hospital without knowing what was wrong the child and on the day she was diagnosed with TB, we were discharged from the hospital”* (IDI_M3P9, female, 25 years, caregiver, child 9 years, completed treatment).

*“When I brought him to the hospital, they gave him an injection and amoxyllin without testing him to find out what it is.”* (IDI_ PLT_M2Q7, female, 28, patient and caregiver, child 2 years, on treatment).

Within these narratives, participant-reported missed opportunities for earlier TB investigation were identified at initial or repeat encounters where persistent, recurrent, or worsening symptoms did not lead to timely TB testing. These descriptions are based on participants’ and caregivers’ accounts of the care received and should not be interpreted as a formal clinical audit of individual healthcare workers’ decisions. Interpreted through Candidacy Theory, participants repeatedly identified a need for care and navigated services, often despite financial, physical, and practical constraints. However, repeated presentation did not consistently result in their being recognized and acted upon as candidates for TB investigation; instead, many were initially channeled into alternative diagnostic or treatment pathways.

#### Economic, psychological, and emotional burden

The prolonged pre-diagnostic period imposed substantial economic, psychological, and emotional burdens on participants and their households. Many described financial strain due to loss of income, repeated transport costs, and reliance on family or friends for support. Illness frequently interrupted livelihoods, with participants reporting halted businesses, lost wages, and increased dependency on relatives. Transport costs to multiple facilities and repeated hospital visits compounded these challenges, particularly for those managing chronic symptoms or caring for children. Some participants described having to walk long distances to access care when funds were unavailable, further highlighting the economic vulnerability induced by delayed diagnosis.

*“I spent a lot of money… I had a business before. But after the illness I lost the business…I used to sell beer wholesale.”* (IDI_A7R6, female, 28, caregiver, child 1 year 9 months, on treatment).

*“I was weak, even in the joints. I was forcing myself to work, so that I find some income to buy food*.” (IDI_PLT_X9A3, male, 28, patient on treatment).

Beyond financial pressures, participants experienced considerable psychological distress. Stress, fear, frustration, and exhaustion emerged as recurring themes, particularly as uncertainty around diagnosis persisted and symptoms worsened. Caregivers experienced heightened anxiety due to responsibility for ill children and the emotional toll of navigating complex healthcare pathways. Unsuccessful care encounters and delays intensified feelings of helplessness, while the necessity to make trade-offs between health and livelihood amplified the burden. For many, economic and emotional challenges were intertwined, with each exacerbating the other over time.

*“During the time when I was told that I should go to QECH, I had nothing in my pocket because by then I had been sick since October, and I had no money… Yes, I always think about what I can do to earn a living, I have a child, and I am concerned about where to get food and how to recover my business…but I am not worried that I have HIV.”* (IDI-N0C3, female, 53, completed treatment).

*“I was also worried about our life as a family, as I am a breadwinner and here in Malawi we worry about what we will eat daily. My biggest concern of all was that I was going to battle with two big diseases.”* (IDI_R8W4, male, 31, completed treatment).

Participants also described anticipatory stress and worry about future consequences, including the sustainability of their businesses, children’s education, and household wellbeing. The compounded burden of prolonged uncertainty, repeated travel, and financial sacrifice often forced families to reprioritize basic needs, such as food and shelter, to accommodate ongoing care. The narratives illustrate how the pre-diagnostic period was experienced as a dynamic interplay of economic hardship and emotional strain, shaping both care-seeking behavior and household resilience.

*“It affected my life so much because at that time…I was failing to go to school.”* (IDI_X4N2, female, 21, patient on treatment).

*“Life was tough for me at that time, and I was thinking that it was just good for me leave the hospital without the doctors’ knowledge…I thought that it could be difficult for me to escape with her in that state.”* (IDI_M3P9, female, 25 years, caregiver, child 9 years, completed treatment).

## Discussion

Our study sought to explore pre-diagnostic experiences of TB patients with particular attention to symptom interpretation, social influences, healthcare access, and burden of disease. The findings provide an in-depth, patient-centered account of how diagnostic delays may rise through repeated engagement with health services in the context of persistent symptoms and diagnostic uncertainty. Patients frequently described seeking help multiple times within primary care setting, yet TB testing was reported to occur only later in the care trajectory or after other treatments had been tried. Our findings are consistent with studies that have previously identified health providers and health systems as potential contributors of diagnostic and treatment delay (13, 33, 34). In contrast, other studies attribute diagnostic delay to factors outside of healthcare (10, 35, 36).

### Reframing diagnostic delay as a process

Our main study contribution is this added perspective; rather than conceptualizing diagnostic delay solely as a measurable interval between symptom onset and treatment initiation, our findings suggests that the diagnostic period for TB unfolds as a dynamic, socially embedded process shaped by symptom interpretation, lay consultation, repetitive engagement with the health system, stigma, and accumulating economic and emotional burden.

By examining and mapping how patients navigate care-seeking trajectories across multiple primary facilities and screening platforms, our findings demonstrate how patient-level and health system-level processes intersect to produce prolonged diagnostic uncertainty. This creates a diagnostic loop where patients repeatedly present to care without reaching diagnostic resolution. From a program perspective, these repeated encounters may represent opportunities for renewed assessment rather than additional episodes of care.

### Theory interpretation

Social cognitive theory and candidacy theory were useful in interpreting our findings. From a Social Cognitive Theory Perspective, symptom interpretation and care-seeking were shaped through observational learning, prior experiences with familiar illness such as malaria, which determined patients’ evaluation of symptom severity and appropriate responses. However, behavior alone does not fully explain diagnostic delay. Candidacy Theory provided a complementary lens by showing how eligibility for TB investigation is socially and institutionally negotiated during healthcare encounters. Our findings indicate that candidacy for TB investigation was not consistently recognized at first contact, despite repeated presentations.

Together, these theories suggest that diagnostic uncertainty was shaped by the interaction between participants’ symptom interpretation and care-seeking, and processes of recognition and response during healthcare encounters. Because this study relied on participant and caregiver accounts, it cannot determine the relative contribution of institutional processes, clinical decision-making, or facility-level constraints to delayed diagnostic resolution.

### Repetitive access and missed diagnostic opportunities within the current architecture of health systems

A defining feature of the current study findings is a culture of repetitive access to care prior to diagnosis of many patients. This aligns with studies showing that some patients had accessed the emergency department at least once prior to a chronic disease diagnosis (37–39). Participants commonly described multiple visits, sometimes five or more, before TB investigations were initiated. These encounters involved symptomatic treatment, alternative diagnoses, temporary improvement and recurrence of symptoms.

Importantly, these encounters did not represent disengagement or indifference; they reflected sustained patient effort within care pathways that did not provide timely diagnostic resolution. These accounts challenge interpretations of “patient delay” that equate delayed diagnosis primarily with late presentation or insufficient care-seeking. Instead, they suggest that diagnostic delay may also arise when repeated healthcare encounters do not result in diagnostic resolution. Because the present study relies on participant accounts and did not independently assess clinical decision-making, diagnostic algorithms, test availability, provider workload, or facility records, these findings should be interpreted as perceived and reported missed diagnostic opportunities rather than independently verified health-system failures.

### The central role of social networks

In this study, care-seeking decisions were rarely made in isolation. Social networks functioned as interpretive communities that shaped symptom recognition, endorsing household remedies including self-medication, and mobilizing escalation to formal care.

Initial responses often involved lay consultations when symptoms were mild and relatively manageable. Yet these same networks often encouraged hospital attendance when illness became visibly severe. Teachers, employers, siblings, and neighbors encouraged referrals and provided transport money or accompaniment. Similar findings were reported in several studies (40–43) that reported social networks as key contributors to the nature of healthcare access individuals decide to adopt, and were occasionally used as a medium of providing health interventions. Thus, social influence should not be viewed as purely delaying or enabling. In addition, delay cannot be reduced to individual cognition. Illness trajectories unfolded within relational contexts that could both sustain and shorten diagnostic pathways.

### Knowledge, fear, and evolving understandings of TB

Participants’ knowledge of TB was heterogeneous and frequently evolved during the illness journey. While many understood TB as airborne and curable, misconceptions persisted, including associations with HIV, moral behavior, or occupational exposure. Notably, knowledge was often acquired after repeated facility encounters rather than shaping early help-seeking.

This temporal dimension of knowledge acquisition suggests that awareness alone is insufficient. Even participants who had heard of TB did not initially recognize their own symptoms as indicative of the disease. Recognition was often triggered by functional decline rather than duration thresholds. This finding highlights the need for community messaging that emphasizes persistent or activity-limiting symptoms and encourages early testing at first presentation.

### Economic precarity and the cost of persistence

A major consequence of repeated healthcare attendance was its cumulative economic impact. Most participants relied on small scale businesses, or daily wage activities that depended on regular physical presence and productivity. As symptoms worsened and healthcare visits increased, participants described declining income, inability to work, and reduced household economic security. Similar economic consequences have been reported across high TB burden settings, where pre-diagnostic costs often accumulate long before treatment begins (44–47).

Repeated health facility visits intensified this burden. Each encounter required transport costs, waiting time, and missed opportunities for income generation. For individuals operating on narrow financial margins, repeated unresolved consultations translated into livelihood erosion, business disruption, and increasing dependence on family support. Several participants described borrowing money for transport, food, or healthcare expenses, resulting in debt that persisted beyond diagnosis.

Importantly, these economic consequences were not solely a result of TB illness itself but were amplified by delayed diagnostic resolution. Patients demonstrated persistence in seeking care, yet repeated missed diagnostic opportunities prolonged both illness and financial hardship. Viewed through this lens, diagnostic delay represents not only a clinical problem but also a process that deepens household vulnerability and redistributes economic burden across family networks. This has implications for how patient-centered TB care is operationalized. Financial protection should consider not only the direct costs of TB diagnosis and treatment, but also the cumulative costs generated by repeated healthcare encounters before diagnostic resolution. Reducing unnecessary repeat visits may be an important form of financial protection in its own right.

### Implications for patient-centered TB care

These recommendations are consistent with the WHO End TB Strategy’s emphasis on integrated, patient-centered care and prevention, including timely access to TB assessment and diagnosis for people with symptoms compatible with TB (7). Our findings suggest that improving TB care requires complementary attention to both delayed presentation and missed opportunities for diagnosis during repeated healthcare encounters. Primary care services could strengthen diagnostic vigilance for patients re-presenting with persistent or worsening symptoms. Where feasible, repeat attendance for unresolved symptoms could serve as a prompt for renewed TB symptom assessment and consideration of TB testing. Such mechanisms could help interrupt the diagnostic loops described by participants, rather than relying on participants’ recognition of worsening symptoms, or repeated care-seeking.

This could be supported through simple facility-level interventions, such as clinically flagging repeated attendance through paper or electronic records and creating clear pathways for escalation when diagnostic uncertainty persists. Depending on feasibility, escalation could entail senior clinical review, immediate TB assessment where available, referral to a facility with diagnostic capacity or to a specialized TB service provider within the same facility. These measures would not imply that every repeat visit represents TB but would create an opportunity for TB and other diagnoses to be considered when symptoms remain unresolved. Further implementation research could assess whether such approaches reduce healthcare encounters, improve continuity of care and the timeliness of TB investigation.

Zimbabwe’s TB Stamp Strategy provides an illustration of a routine-care prompt intended to support earlier identification and investigation of TB among people seeking healthcare (48). Although this approach was developed for a different context and was not evaluated in the present study, its underlying principle is relevant to the diagnostic loops described by participants; routine clinical encounters can be used as opportunities to reconsider TB when symptoms remain unresolved. Any adaptation of a similar flagging mechanism in Malawi should be aligned with national guidelines, testing capacity, and should be evaluated for feasibility, acceptability, and its effect on timely diagnosis.

Community-based TB screening should also be linked more closely with primary healthcare services. Where community screening identifies individuals requiring further assessment or referral, clear mechanisms for referral, documentation and follow-up could help ensure continuity between community and facility-based diagnostic pathways. This is particularly relevant where patients move between community screening platforms and primary healthcare facilities during their search for a diagnosis.

The findings further suggest that community communication should complement awareness of TB symptoms with clearer guidance on when and how to seek further care. Participants did not always initially interpret their symptoms as TB-related, and recognition was sometimes associated with persistent symptoms or declining ability to perform usual activities. Community messaging could therefore emphasize persistence or worsening of any symptoms and functional decline, alongside encouragement to seek or return for assessment when symptoms do not resolve with initial treatment.

The diagnostic loop identified in this study may also offer a potential quality-improvement lens for primary care. Rather than measuring diagnostic delay only as the elapsed time from symptom onset to treatment initiation, programs could explore whether repeated attendance for unresolved symptoms can be used to identify potential missed diagnostic opportunities. Implementation research could examine feasible indicators such as the number of healthcare encounters before diagnostic resolution, the proportion of patients with repeated unresolved symptoms who receive repeat TB assessment, and the time between repeated presentation and appropriate TB investigation. Such measures would require prospective evaluation before being considered for routine program monitoring.

Finally, repeated healthcare attendance generated cumulative transport costs, waiting time, lost income and reliance on household support. From a patient-centered perspective, reducing avoidable repeat visits may itself contribute to financial protection. Where appropriate and feasible, decentralized diagnostic services, patient navigation, and targeted transport or social-protection support could reduce the economic burden associated with repeated attempts to obtain a diagnosis. Such interventions should complement, rather than substitute for, efforts to improve timely diagnostic resolution within primary care.

### Strengths and limitations

This study benefits from inclusion of diverse participants across multiple urban primary care settings and diagnostic platforms, enabling a nuanced understanding of pre-diagnostic trajectories. The use of in-depth interviews conducted in participants’ preferred environments allowed rich reflection on lived experience. A hybrid deductive–inductive analytic approach enhanced rigor and responsiveness to emergent themes.

However, this study has several limitations. First, the study was conducted in urban Blantyre and may not reflect pre-diagnostic TB experiences in rural Malawi, where travel distances, service availability, economic conditions, and health literacy may differ. Second, interviews were conducted after TB diagnosis, and accounts of symptom onset, care-seeking, and earlier healthcare encounters may have been affected by recall bias. We sought to minimize this risk by including participants at different stages of TB care, including individuals receiving treatment soon after diagnosis and individuals who had completed treatment. Third, participants were recruited through TB officers and had successfully entered TB care. Although researchers clarified their independence from facility staff and that participation would not affect healthcare, accounts may have been influenced by social desirability or by concerns about ongoing relationships with services. Consequently, individuals who disengaged from care, could not be contacted, or never reached formal services were not represented, and the most severe forms of access failure may be underrepresented. Finally, because the analysis relied on participant and caregiver accounts rather than facility records, provider interviews, diagnostic registers, or observation of clinical encounters, we could not independently assess clinical decision-making, diagnostic capacity, or the appropriateness of individual clinical decisions.

## Conclusion

This study demonstrates that participants with TB in urban Malawi described persistent engagement with healthcare alongside diagnostic uncertainty. Participants and caregivers reported repeatedly seeking care, mobilizing social networks, and enduring financial and emotional strain in pursuit of answers. These accounts suggest that opportunities to improve timely diagnosis may exist not only before the first healthcare encounter, but also within subsequent encounters when symptoms remain unresolved.

A patient-centered response should therefore address both access to care and the responsiveness of services once people enter the healthcare system. Strengthening reassessment of persistent symptoms, recognizing repeated healthcare attendance as a potential prompt for renewed TB consideration, improving linkages between community and facility-based diagnostic services, and reducing the financial burden of repeated healthcare visits may help reduce avoidable diagnostic uncertainty. These approaches should be evaluated within routine primary care to determine their feasibility, acceptability and effect on timely TB diagnosis.

## Data Availability

The raw data supporting the conclusions of this article will be made available by the authors, without undue reservation.
